# Reporters Transiently Transfected into Mammalian Cells Are Highly Sensitive to Translational Repression Induced by dsRNA Expression

**DOI:** 10.1371/journal.pone.0087517

**Published:** 2014-01-27

**Authors:** Jana Nejepinska, Radek Malik, Susan Wagner, Petr Svoboda

**Affiliations:** 1 Institute of Molecular Genetics of the ASCR, v.v.i., Prague, Czech Republic; 2 Institute of Microbiology of the ASCR, v.v.i., Prague, Czech Republic; The John Curtin School of Medical Research, Australia

## Abstract

In mammals, double-stranded RNA (dsRNA) can mediate sequence-specific RNA interference, activate sequence-independent interferon response, or undergo RNA editing by adenosine deaminases. We showed that long hairpin dsRNA expression had negligible effects on mammalian somatic cells—expressed dsRNA was slightly edited, poorly processed into siRNAs, and it did not activate the interferon response. At the same time, we noticed reduced reporter expression in transient co-transfections, which was presumably induced by expressed dsRNA. Since transient co-transfections are frequently used for studying gene function, we systematically explored the role of expressed dsRNA in this silencing phenomenon. We demonstrate that dsRNA expressed from transiently transfected plasmids strongly inhibits the expression of co-transfected reporter plasmids but not the expression of endogenous genes or reporters stably integrated in the genome. The inhibition is concentration-dependent, it is found in different cell types, and it is independent of transfection method and dsRNA sequence. The inhibition occurs at the level of translation and involves protein kinase R, which binds the expressed dsRNA. Thus, dsRNA expression represents a hidden danger in transient transfection experiments and must be taken into account during interpretation of experimental results.

## Introduction

Double-stranded RNA (dsRNA) is a unique structure with important biological effects. Viruses often give rise to dsRNA during their life cycle; therefore, dsRNA is recognized by a vertebrate cell as a hallmark of viral presence (reviewed in [1]). dsRNA can also arise endogenously in a cell, being formed upon basepairing between complementary transcripts or by intramolecular pairing within a transcript, thus forming a hairpin. In mammalian cells, dsRNA can enter three pathways: RNA interference (RNAi), RNA editing, and the interferon response. RNAi mediates sequence-specific RNA degradation guided by ∼22 nt small interfering RNAs (siRNAs) produced from long dsRNA by RNase III Dicer (reviewed in [2]). RNA editing is mediated by the adenosine deaminase acting on RNA (ADAR) family of enzymes. ADARs are nuclear and cytoplasmic enzymes activated by dsRNA that convert adenosines to inosines (which are recognized as guanosines during translation). Editing of dsRNA can cause target RNA degradation or modify its coding potential (reviewed in [3]). The interferon response is a complex network of vertebrate pathways involved in the innate immune response against viruses (reviewed in [4]). One of the key factors in the interferon response is protein kinase R (PKR), which is activated upon binding of dsRNA to its dsRNA-binding domain. Activated PKR phosphorylates the α-subunit of the eukaryotic initiation factor 2 (eIF2α), which stabilizes the GEF-eIF2-GDP complex and, consequently, causes the inhibition of translation initiation (reviewed in [5]). In addition to PKR, the interferon response involves coordinated action of other molecules, such as oligoadenylate synthetase, RNase L, RIG-I, or NF-κB [1]. The inhibition of proteosynthesis by PKR is sequence-independent and typically affects translation in general [5]. Nevertheless, several groups observed restricted PKR effects and selective inhibition of specific mRNAs [6], [7].

To examine the fate of long dsRNA synthesized in the nucleus, we previously expressed dsRNA as a long hairpin located in the 3′UTR of an EGFP reporter [8]. We showed that mammalian cells can tolerate dsRNA expression; dsRNA neither activated the interferon response nor induced RNAi in somatic cells [8]. However, we noticed sequence-independent suppression of luciferase reporters in transient co-transfection experiments when a dsRNA-expressing plasmid was present. This observation was complemented by an independent study of RNAs produced by transiently transfected plasmids, which revealed that some common plasmids can produce dsRNA and suppress co-transfected reporters [9].

Transient co-transfection is a common approach to deliver an experimental plasmid together with appropriate reporters into mammalian cells. A dual luciferase reporter system is among the most common reporter systems as it allows for using one luciferase as a targeted experimental reporter and the other one as a non-targeted control for normalization. Here, we systematically explored reporter expression in co-transfections experiments where one of the co-transfected plasmids produces dsRNA. We show that transient co-transfection of a dsRNA-expressing plasmid inhibits co-transfected reporter plasmids in a sequence-independent manner. The effect is posttranscriptional, involves translational repression, and is PKR dependent. Remarkably, this dsRNA response strongly affects expression originating from co-transfected plasmids but neither the expression of endogenous genes nor stably integrated reporters. Our data suggest that, upon appearance of dsRNA in a transient transfection, PKR elicits a selective translational repression of mRNAs from co-transfected plasmids. This effect may represent a distinct mode of PKR activity as it can appear without the typical interferon response, such as the activation of NF-κB and interferon-stimulated genes. In any case, our results provide an important framework for the correct interpretation of experiments based on transient transfections.

## Materials and Methods

### Plasmids

**Figure 1:**
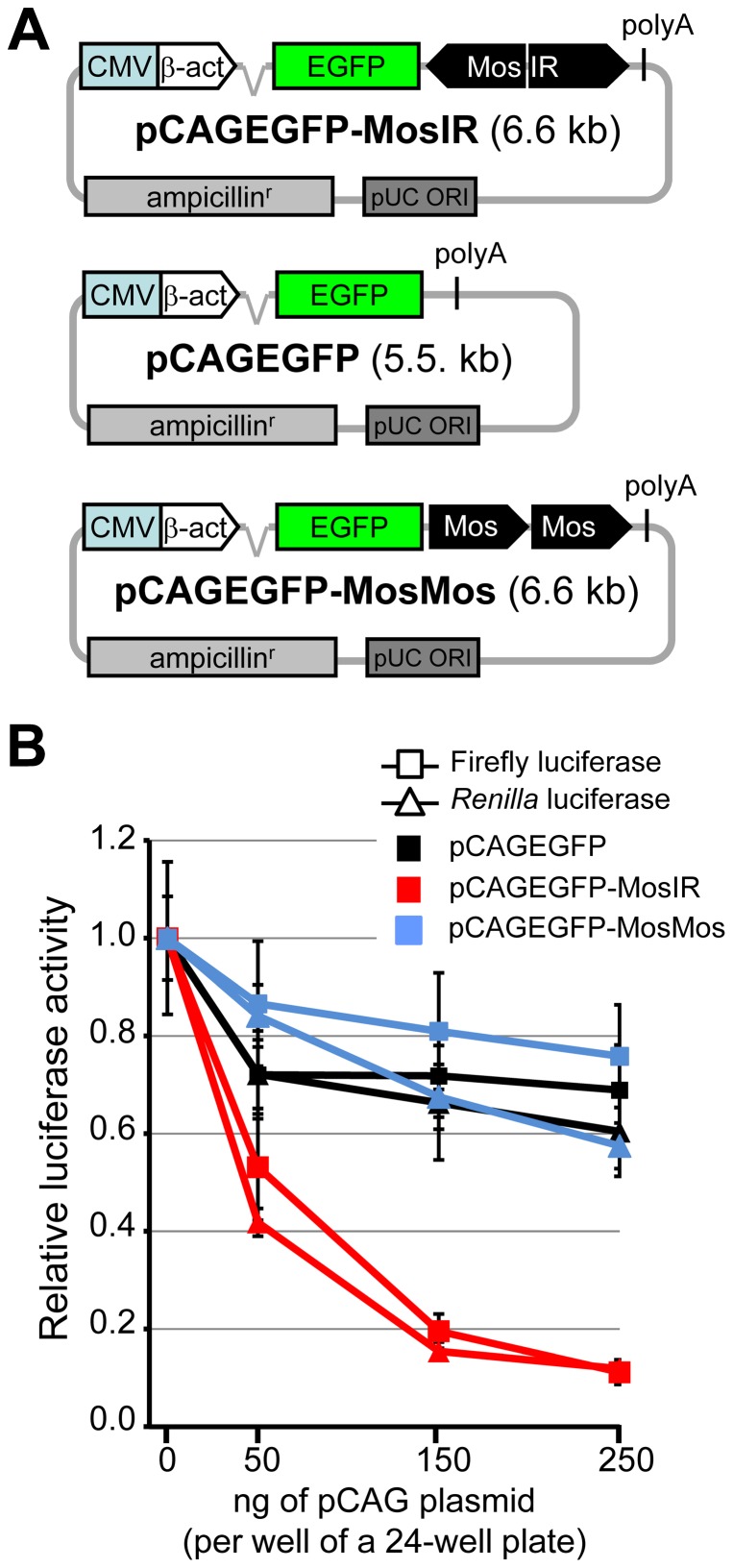
Expression of hairpin RNA inhibits the luciferase activity of transiently transfected reporter plasmids. (A) Schematic composition of pCAGEGFP-MosIR, pCAGEGFP, and pCAGEGFP-MosMos plasmids. (B) Reporter activity is inhibited by hairpin RNA in a concentration-dependent manner. HEK-293 cells were transiently transfected with a constant amount of firefly luciferase (square), *Renilla* luciferase (triangle) reporter plasmids, and increasing amount of a tested plasmid. Luciferase activities were measured 48 hours post-transfection. pBluescript was added to maintain a constant amount of transfected DNA. Both luciferase activities are shown relative to cells transfected with 0 ng of the pCAGEGFP-MosIR. Data are shown as an average of at least 3 experiments made in triplicates. Error bars  =  SEM.

Schematic structures of the relevant parts of plasmid constructs used in the project are shown in Fig. 1A and described in the text. Plasmids were purchased from the manufacturers specified in parentheses: pBluescript II KS(+) (Stratagene), pGL4-SV40 (Promega; for simplicity referred to as FL) and phRL-SV40 (Promega; for simplicity referred to as RL). The construction of plasmids pCAGEGFP-MosIR [8] and pCAGEGFP [10] was described previously. ZP3EGFP-Lin28IR (Flemr *et al.*, unpublished) and ZP3EGFP-Elavl2IR (Chalupnikova *et al.*, unpublished) plasmids containing mouse *Lin28a/b* and *Elavl2* sequences, respectively, were constructed similarly to pCAGEGFP-MosIR plasmid and will be described in detail elsewhere. For the construction of pCAGEGFP-Lin28IR and pCAGEGFP-Elavl2IR plasmids, parental pCAGEGFP plasmid was modified by inserting a NotI site downstream of the EGFP coding sequence and Lin28IR and Elavl2IR fragments were cloned into the NotI site. pCAGEGFP-MosMos plasmid was produced by inserting a *Mos* fragment (the same as in pCAGEGFP-MosIR) into a BglII site of pCAGEGFP plasmid; screening of was subsequently performed to identify a plasmid containing a head-to-tail insertion of two *Mos* fragments. All plasmids were verified by sequencing.

### Cell culture and transfection

Human HEK-293, HeLa cells, and 3T3 were maintained in DMEM (Sigma) supplemented with 10% fetal calf serum (Sigma), penicillin (100 U/mL, Invitrogen), and streptomycin (100 µg/mL, Invitrogen) at 37°C and 5% CO_2_ atmosphere. For transfection, cells were plated on a 24-well plate, grown to 50% density and transfected using TurboFect *in vitro* Transfection Reagent (Thermo Scientific), unless stated otherwise. For Nanofectin and calcium phosphate transfection, cells were plated on a 24-well plate, grown to 70% density and transfected using Nanofectin (PAA) reagent according to the manufacturer's protocol. Calcium phosphate transfection was performed according to a standard protocol [11]. For polyethyleneimine (PEI) tranfection, 28 µg of DNA were used per 15 cm dish, PEI to DNA ratio was 6∶1.

For 24-well plates, cells were co-transfected with 100 ng of each FL and RL reporter plasmids and various amounts of a tested plasmid (50–250 ng per well). The total amount of transfected DNA was kept constant (700 ng/well) by adding promoterless pBluescript or parental pCAGEGFP plasmid. After 48 hours, cells were washed with phosphate-buffered saline (PBS) and lysed with Passive Lysis Buffer (Promega). Luciferase reporter activity was assessed using the Dual-Luciferase Reporter Assay (Promega) and luminiscence intensity was measured by Modulus Microplate Multimode Reader (Turner Biosystems). Variability in cell densities was minimized by normalization to total protein concentration measured by Protein assay kit (Bio-Rad) according to the manufacturer's protocol.

Transfection efficiency of transiently-transfected cells was routinely 70% or more (estimated by microscopy and FACS). FACS and microscopy analysis of transiently co-transfected fluorescent protein-expressing reporters showed that co-transfected reporters were typically co-expressed and the level of expression of reporters correlated in individual cells as well as in FACS-sorted population of cells.

HEK-293 cells stably expressing both RL and FL reporters were described elsewhere [8]. A similar procedure was used for establishing RL-only-expressing stable HEK-293 cells. HeLa PKR knock-down cells were generated by a similar procedure except that Zeocin (250 µg/ml) was used for the selection of positive clones. shRNA (5′-GATCCCCGGCAGTTAGTCCTTTATTATTCAAGAGATAATAAAGGACTAACTGCCTTTTTA-3′) targeting PKR was cloned into pTER plasmid [12]. A stable cell line expressing empty pTER plasmid was used as a control. The selected clones were screened for PKR knockdown by western blotting.

### Flow cytometry

HEK-293 cells plated in 24-well plates were co-transfected with 150 ng/well of RFP reporter plasmid (pCI-RFP) and 350 ng/well of either pCAGEGFP-MosIR or pCAGEGFP plasmid. Cells were collected 24 and 36 hours post-transfection and analyzed by flow cytometry using LSRII cytometer (BD Bioscience). Data analysis was performed by FlowJo software (Treestar, Inc.).

### High-throughput sequencing (SOLiD)

HEK-293 cells were plated on 6-well plates and grown to 50% density. Cells were transfected with 2.7 µg/well of either pCAGEGFP or pCAGEGFP-MosIR plasmid, cultured for 48 hours, washed with PBS, and total RNA was isolated using RNAzol (MRC) according to the manufacturer's protocol. RNA quality was verified by Agilent 2100 Bioanalyzer. The library construction from total RNA and high-throughput sequencing of the RNA transcriptome were performed by Seqomics (Szeged, Hungary) using SOLiD (version 4.0) sequencing platform. Bioinformatic analysis was performed as described previously [8]. High throughput sequencing data in color-space format were deposited in the GEO database (GSE46959).

### Western blotting

HEK-293 cells were grown in 6-well plates. Before collection, cells were washed with PBS and lysed in Whole-cell lysis buffer (10% glycerol, 0.5 mM EDTA, 1 mM DTT, 2 mM natrium fluoride, 0.2% Triton X-100 in PBS pH 7.4) supplemented with protease and phosphatase inhibitors (Calbiochem). Proteins were separated on 10% polyacrylamide gel and transferred to PVDF membrane (Millipore). The following antibodies were used for detection: total PKR (Abcam #ab32052, 1∶5000 dilution), PKR [pT^446^] (Abcam #ab32036, 1∶1000), total EIF2α (Santa-Cruz #sc-11386, 1∶1000), EIF2α [pS^52^] (Life Techonologies #44728G, 1∶1000), RPS14 (Santa-Cruz #sc-68873, 1∶1000), and tubulin (Sigma #T6074, 1∶5000). SuperSignal West Femto Chemiluminescent Substrate (Pierce) was used for detection.

### Polysome profiling analysis

Cells were plated on 15-cm dishes, grown to 50% density and co-transfected with FL (8 µg per dish), RL (8 µg per dish), and either pCAGEGFP or pCAGEGFP-MosIR plasmid (12 µg per dish) using polyethyleneimine. After 24 hours, cells were harvested with PBS in the presence of cycloheximide (100 µg/ml). Whole-cell extracts were prepared in breaking buffer (20 mM Tris-HCl, pH 7.5, 50 mM KCl, 10 mM MgCl_2_, 10% glycerol, 1% Triton X-100, 1 mM dithiothreitol, 1 mM phenylmethylsulfonyl fluoride, Protease Inhibitor Cocktail Set III (Calbiochem), RiboLock RNase inhibitor (Thermo Scientific) (0.3 U/µl)). Ten or fifteen A_260_ units of whole-cell extracts were separated by velocity sedimentation on a 5% to 45% sucrose gradient by centrifugation at 39,000 g for 2.5 h in SW41Ti rotor (Beckman). Gradient fractions were collected and scanned at 254 nm to visualize ribosomal species.

For RNA isolation, each fraction was mixed with 1 ml of ice cold 96% ethanol, 3 M sodium acetate (20∶1), and 1 µl of glycogen (RNA grade, Thermo Scientific) and incubated overnight at −20°C. Samples were centrifuged (16,000 g, 30 min, 4°C) and RNA from pellets was isolated using RNAzol (MRC) according to the manufacturer's protocol. Residual DNA was degraded by Turbo DNase (Ambion) and the same portion of each fraction was reverse-transcribed using RevertAid Premium Reverse Transcriptase (Thermo Scientific) according to the manufacturer's instructions. Reverse transcriptase was omitted in control (-RT) samples. Real-time PCR was performed on LC480 machine (Roche) using Maxima SYBR green qPCR Master Mix (Thermo Scientific). Primers sequences a provided in Table S1. Values of crossing points were evaluated and corrected according to PCR efficiency for each reaction. For protein isolation, each fraction was ethanol precipitated according to [13]. The equivalent portion of each fraction was used for SDS-PAGE and western blotting.

### RNA immunoprecipitation

HEK-293 cells (∼10×10^6^ cell/dish) were transfected by pCAGEGFP or pCAGEGFP-MosIR plasmids using PEI and grown in 10 cm dishes. After 48 hours, cells were washed with PBS and scraped into IP buffer (20 mM HEPES, 1 mM EDTA, 0.1 M NaCl, 0.5% NP-40 (Nonidet), 1 mM DTT, 0.2 mM Na_3_VO_4_, 50 mM NaF, 1X Protease inhibitor cocktail set (Calbiochem), 1X Phosphatase inhibitor cocktail set (Calbiochem), and RiboLock RNase Inhibitor (Thermo Scientific)) and incubated on ice for 15 minutes with occasional vortexing. Lysates were passed several times through 21G and 27G needles and incubated on ice for 15 minutes with occasional vortexing. Samples were centrifuged for 20 minutes at 12,000 g and 4°C and the supernatant was diluted 5X with IP buffer without NP-40. Samples were mixed with Protein-A-Sepharose beads 4B Fast Flow (Sigma) and 5 µg of either PKR antibody (Abcam #ab32052) or control IgG antibody (Abcam #37415) and incubated for 2 hours at 4°C on a rotator. Samples were washed 5X with IP buffer with 0.1% NP-40, centrifuged for 4 minutes at 4000 g and 4°C. RNA was isolated from the pellet using RNAzol. RNA was analyzed by real-time PCR. Primers sequences a provided in Table S1.

## Results

### dsRNA expressing plasmid inhibits co-transfected reporters

Our previous results suggested that dsRNA originating from a plasmid suppresses the expression of co-transfected reporters in a sequence-independent manner [9]. To examine the phenomenon, we used dsRNA-expressing pCAGEGFP-MosIR plasmid, in which the inverted repeat of the *Mos* gene sequence (MosIR) is inserted into the 3′UTR of an EGFP reporter controlled by a strong chimeric (CMV/β-actin) promoter (Fig. 1A) [8]. The plasmid-derived transcript forms an intramolecular duplex (∼500 bp) downstream of the EGFP coding sequence. *Mos* hairpin is one of the most studied long dsRNAs [8], [14]–[17]; its formation of dsRNA structure has been demonstrated both *in vitro* [17] and *in vivo* [8]. *Mos* expression and function are restricted to oocytes [18], [19]; hence, effects observed in other cell types are sequence-independent. pCAGEGFP-MosIR plasmid does not induce efficient RNAi in cultured cells, presumably because of inefficient processing into siRNAs [8].

For the initial characterization of pCAGEGFP-MosIR inhibitory effects, we co-transfected HEK-293 cells with a constant amount of *Renilla* luciferase (RL) and firefly luciferase (FL) reporters and with increasing amounts of pCAGEGFP-MosIR plasmid. As a control, we used the parental pCAGEGFP lacking the inverted repeat or pCAGEGFP-MosMos, in which the Mos sequences were inserted as a tandem repeat in a head-to-tail orientation to produce a plasmid with the same size and sequence composition as pCAGEGFP-MosIR but not producing hairpin dsRNA (Fig. 1A). To maintain a constant amount of transfected DNA, we used promoterless pBluescript, which was shown previously to have a minimal impact on co-transfected reporters [9].

pCAGEGFP-MosIR caused a strong (up to 90%) concentration-dependent decrease in both luciferase activities while the inhibitory effect of the two control plasmids was small (Fig. 1B). The observed suppression was presumably sequence-independent because *Mos* sequence lacks similarity to luciferase reporters. pCAGEGFP and pCAGEGFP-MosMos effects provided a good baseline for estimating the impact of transcribed inverted repeat on co-transfected luciferase reporters. A minor reduction of luciferase activities caused by co-transfected pCAGEGFP or pCAGEGFP-MosMos was not surprising because we have previously reported that plasmids affect the expression of co-transfected reporters to various degrees. This phenomenon may be associated with the complex transcription of transfected plasmids, which can be an unexpected source of dsRNA [9].

**Figure 2:**
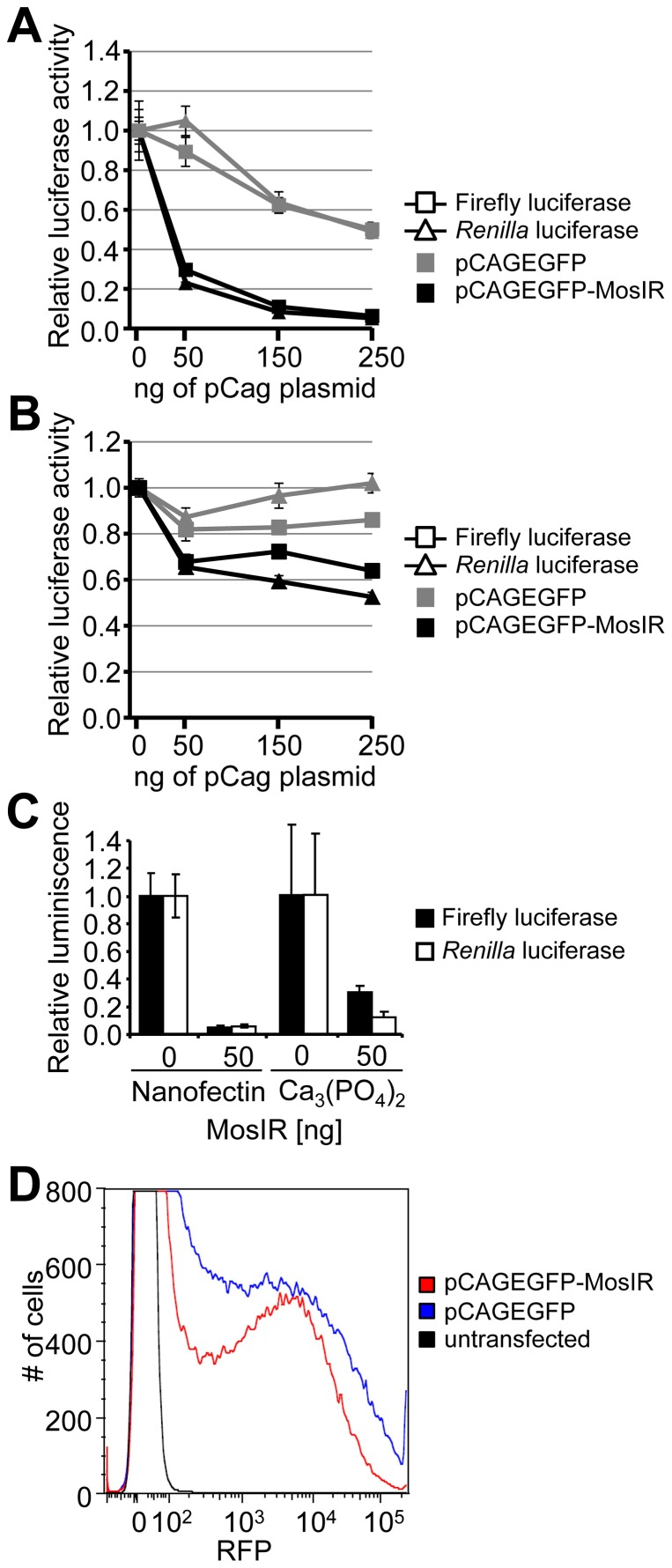
Suppression of co-transfected reporters in mammalian cells is general. (A, B) Suppression of co-transfected reporters in HeLa cells (A) and mouse 3T3 cells (B). Cells were transiently transfected with a constant amount of firefly luciferase (square), *Renilla* luciferase (triangle) reporter plasmids, and increasing amounts of pCAGEGFP-MosIR or pCAGEGFP. Luciferase activities were measured 48 hours post-transfection. pBluescript was added to maintain a constant amount of transfected DNA. Both luciferase activities are shown relative to cells transfected with 0 ng of the pCAGEGFP-MosIR. Data are shown as an average of at least 3 experiments performed in triplicates. Error bars  =  SEM. (C) Suppression of co-transfected reporters is independent of the transfection method. HEK-293 cells were transiently transfected with a constant amount of firefly luciferase (black bars), *Renilla* luciferase (white bars) reporter plasmids, and 50 ng of pCAGEGFP-MosIR using Nanofectin or calcium phosphate transfection. Luciferase activities were measured 48 hours post-transfection. pBluescript was added to maintain a constant amount of transfected DNA. Both luciferase activities are shown relative to cells transfected with 0 ng of the pCAGEGFP-MosIR. Data show a typical experiment measured in triplicates. Error bars  =  SEM. (D) Suppression of a co-transfected RFP reporter. HEK-293 were transiently transfected with 150 ng of RFP reporter plasmid and 350 ng of pCAGEGFP or pCAGEGFP-MosIR. Shown is FACS analysis of RFP fluorescence 36 h post-transfection. The experiment was performed three times, a representative result is shown.

Altogether, our results showed that the inverted repeat inserted into pCAGEGFP induced suppression of co-transfected luciferase reporters. This phenomenon was also observed in human HeLa cells (Fig. 2A) and mouse 3T3 cells (Fig. 2B), although the effect of dsRNA expression in 3T3 cells was weaker. The inhibition was independent of transfection procedure or transfection reagent; it was present also when Nanofectin transfection reagent or calcium phosphate transfection were used (Fig. 2C). To test whether the observed inhibition was specific to luciferase reporters, we co-transfected an RFP-expressing plasmid with either pCAGEGFP or pCAGEGFP-MosIR plasmid and analyzed RFP fluorescence using flow cytometry. Reduced RFP expression in cells co-transfected with pCAGEGFP-MosIR plasmid (Fig. 2D) compared to pCAGEGFP- transfected cells suggests that the inhibition of reporter activity is independent of the co-transfected reporter type.

### Detection of dsRNA expression using high-throughput sequencing (HTS)

**Figure 3:**
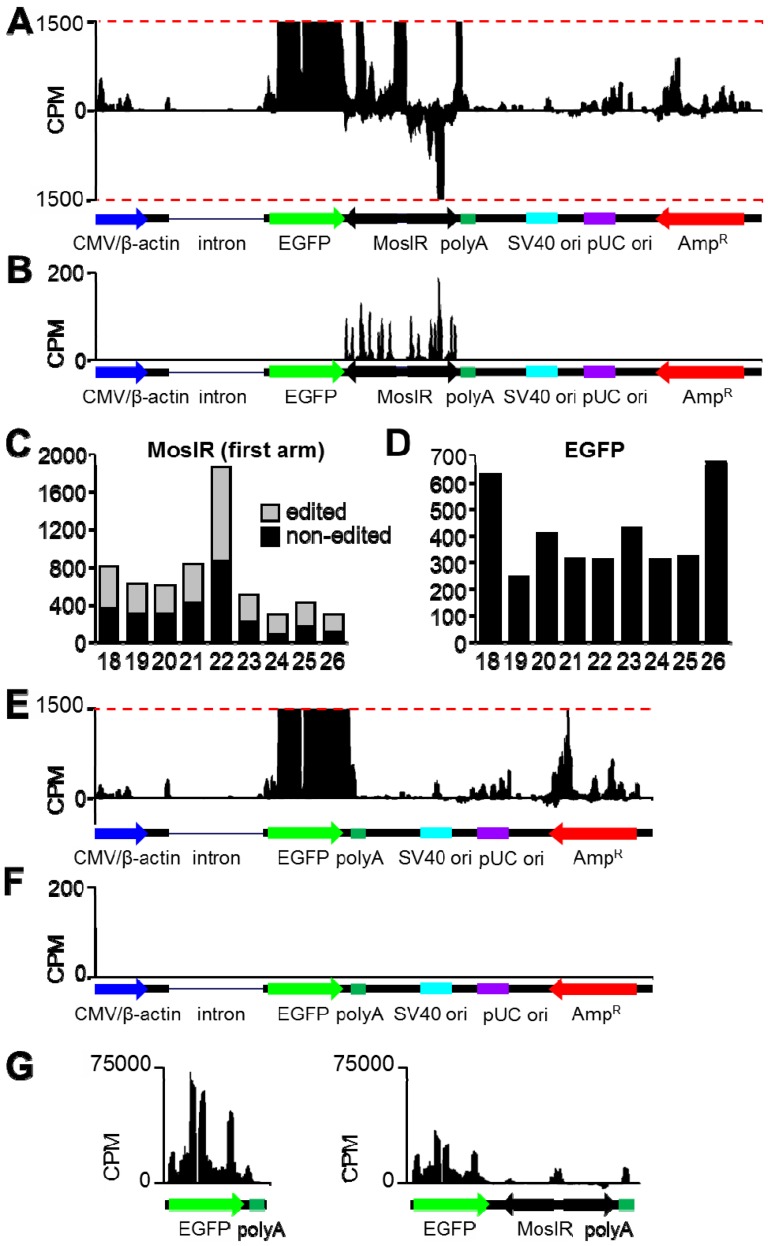
High-throughput sequencing analysis of total RNAs derived from pCAGEGFP and pCAGEGFP-MosIR plasmids. (A) Distribution of 18–50 nt reads that perfectly map to pCAGEGFP-MosIR. Reads mapping to the sense and antisense strands are shown in the upper and lower half of the graph, respectively. Schematic representation of plasmid features is shown below the histogram. The Y-scale represents normalized read density (counts per million, CPM); y-scale maximum corresponds to 1500 CPM to better visualize RNAs produced from the plasmid backbone. Expression from the CMV/β-actin promoter, which yields normalized read density highly exceeding 1500 CPM, can be seen in the panel G. (B) Distribution of reads 20–24 nt in length with up to 5 adenosine-to-guanosine mismatches that map to pCAGEGFP-MosIR. The Y-scale represents normalized read density (counts per million, CPM); y-scale maximum corresponds to 200 CPM. Schematic representation of plasmid features is shown below the histogram. (C, D) Length distribution of edited (gray) and non-edited (black) reads derived from MosIR (C) or EGFP (D) region of pCAGEGFP-MosIR. (E) Distribution of 18–50 nt reads that perfectly map to pCAGEGFP. Description as in the panel a. (F) Distribution of reads 20–24 nt in length with up to 5 adenosine-to-guanosine mismatches that perfectly to pCAGEGFP. Description as in the panel B. (G) Comparison of normalized read density for pCAGEGFP and pCAGEGFP-MosIR transcripts transcribed from the CMV/β-actin promoter. The Y-scale represents normalized read density (counts per million, CPM); y-scale maximum corresponds to 75000 CPM. Schematic representation each transcript is shown below each histogram.

To analyze transcripts arising from pCAGEGFP-MosIR in depth, we performed HTS of HEK-293 cells transfected with either pCAGEGFP or pCAGEGFP-MosIR using SOLiD technology (Fig. 3). The plasmid backbone yielded similar coverage patterns in both plasmids (Fig. 3A and 3E), while the number of sequence tags derived from the EGFP coding sequence of pCAGEGFP-MosIR was approximately three times lower compared to pCAGEGFP (Fig. 3G). To confirm that pCAGEGFP-MosIR produces dsRNA, we analyzed adenosine deamination of short SOLiD sequence tags as previously described [8], [9]. Consistently with previous results, RNA editing of adenosine manifested as adenosine/guanosine (A/G) conversion in sequence tags clustering to the MosIR region (Fig. 3B and 3F). Quantitative analysis of A/G conversion revealed robust editing of small RNAs within the MosIR region (Fig. 3C). On the other hand, A/G conversion was negligible in small RNA reads mapping to the EGFP region of the same plasmid (Fig. 3D). These results are consistent with our previous data [8], [9] and confirm that pCAGEGFP-MosIR generates MosIR dsRNA.

### Sequence independent dsRNA-mediated repression of co-transfected reporters

**Figure 4:**
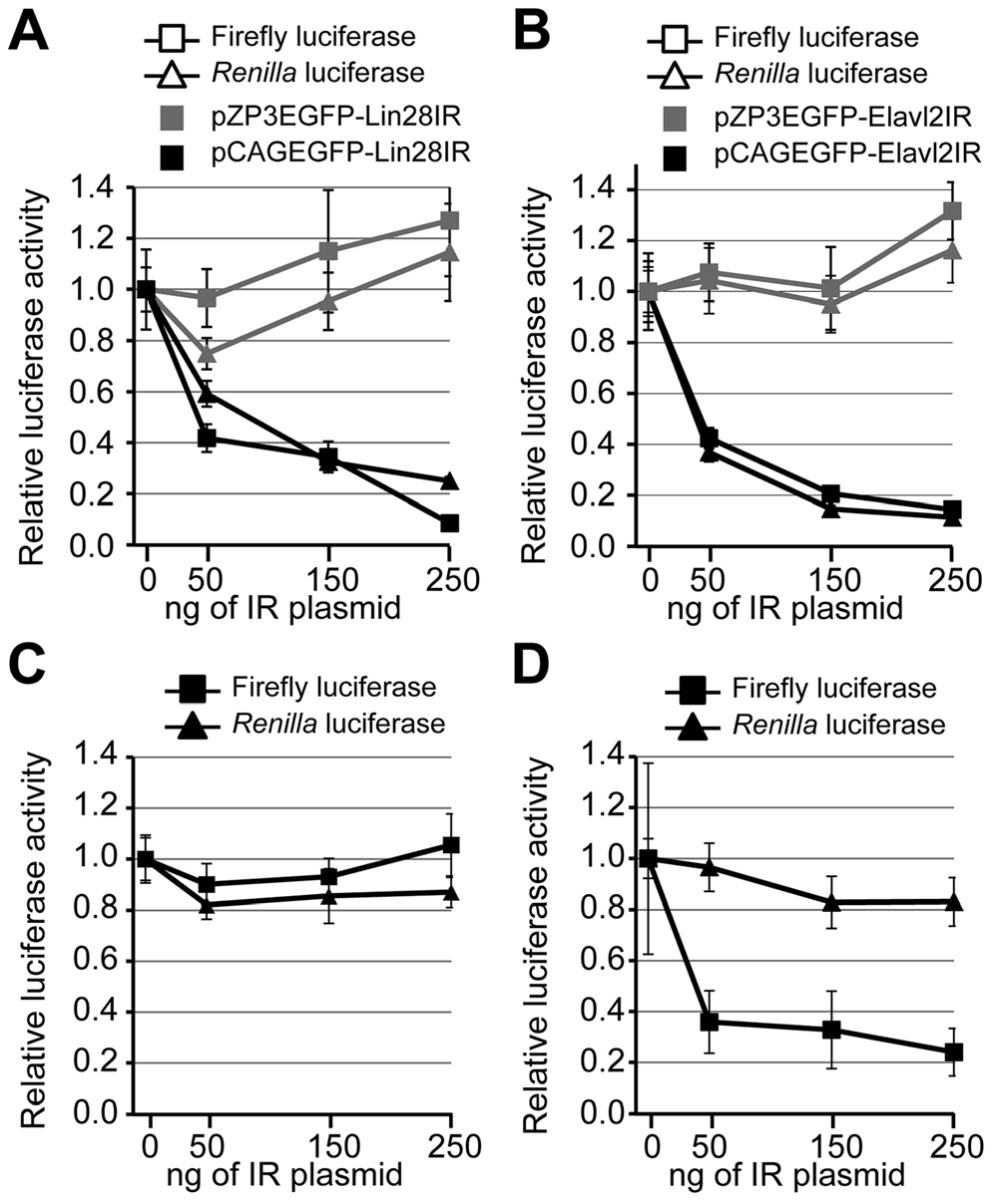
Transiently transfected luciferase reporters are inhibited by expressed dsRNA. (A) The inhibition of reporter activity is independent of the hairpin RNA sequence and it is absent when the inverted repeat is placed downstream of non-active ZP3 promoter. HEK-293 cells were transiently transfected with 100 ng/well of each RL (triangle) and FL (square) reporter plasmids and increasing amount (0–250 ng/well) of either pCAGEGFP-Lin28IR (containing an active promoter) or pZP3EGFP-Lin28IR (containing an inactive promoter). Luciferase activity was analyzed 48 hours after transfection. pBluescript was added to maintain the amount of transfected DNA constant. Both luciferase activities are shown relative to cells transfected with 0 ng of the hairpin-expressing plasmid. (B) Similar to (A) except Elavl2IR-expressing plasmids (pCAGEGFP- Elavl2IR or pZP3EGFP-Elavl2IR) were used. Data are shown as an average of at least 3 experiments made in triplicates. Error bars  =  SEM. (C, D) A hairpin-expressing pCAGEGFP-MosIR plasmid affects the luciferase activity of transiently transfected but not stably integrated reporter plasmids. HEK-293 cells with stably integrated RL and FL reporters (C), or HEK-293 cells with stably integrated RL reporter only (D) were transiently transfected with an increasing amount of pCAGEGFP-MosIR and a constant amount of FL reporter (if not stably integrated). pBluescript was added to maintain the amount of transfected DNA constant. Both FL (squares) and RL (triangles) luciferase activities were analyzed 48 hours post-transfection. Luciferase activities in cells transfected with 0 ng of the pCAGEGFP-MosIR were set to one. Data show an average of at least 3 experiments done in triplicates. Error bars  =  SEM.

To test whether the MosIR-mediated inhibition is dsRNA sequence-independent, we used two additional hairpin sequences. Inverted repeats made of mouse *Lin28a/b* and *Elavl2* sequences were inserted into the same position in pCAGEGFP as the MosIR hairpin and the resulting plasmids pCAGEGFP-Lin28IR and pCAGEGFP-Elavl2IR were co-transfected with luciferase reporters in HEK-293 cells. dsRNA expression from pCAGEGFP-Lin28IR and pCAGEGFP-Elavl2IR was confirmed using RNase T1 treatment and RNA immunoprecipitation with dsRNA-specific antibody (Fig. S1). Plasmids in which the CMV/β-actin promoter was replaced with ZP3 promoter (pZP3EGFP-Lin28IR and pZP3EGFP-Elavl2IR) were used to test the dependence on hairpin expression and served as negative controls. As the ZP3 promoter is oocyte-specific, dsRNA should not be produced when pZP3EGFP-Lin28IR or pZP3EGFP-Elavl2IR are transfected into HEK-293 cells. Similarly to pCAGEGFP-MosIR, pCAGEGFP-Lin28IR and pCAGEGFP-Elavl2IR caused a concentration-dependent decrease in both luciferase activities (Fig. 4A, B). At the same time, co-transfection of ZP3 derivatives did not show an inhibitory effect, confirming that the inhibition depends on dsRNA expression (Fig. 4A, B). Taken together, dsRNA produced from a transiently transfected plasmid has a general, concentration-dependent, and sequence-independent inhibitory effect on the expression of co-transfected reporters.

### dsRNA expression does not suppress stably integrated reporters

The inhibitory effect of *Mos* dsRNA on co-transfected reporters was surprising because our previous analysis revealed only minor changes in endogenous gene expression [8]. Transient transfection of pCAGEGFP-MosIR showed minor effects on the cellular transcriptome (GSE27316) and did not activate the interferon response (monitored by RT-PCR analysis of *IL-8* transcript (Fig. S2)); transfected cells had normal proliferation rate and morphology [8]. In contrast to transient transfections, we did not observe inhibition of RL and FL luciferase reporters integrated in the genome (Fig. 4C). This suggested that the silencing phenomenon particularly concerns expression of transiently transfected reporters. To test this hypothesis, we created a HEK-293 cell line with a stable integration of RL reporter. This cell line was co-transfected with FL reporter and an increasing amount of pCAGEGFP-MosIR plasmid. Consistent with previous results, FL activity was strongly reduced in a concentration-dependent manner while RL activity remained unaffected (Fig. 4D). Transiently-transfected reporters likely produce higher numbers of reporter mRNA molecules per cell compared to integrated reporters. To test whether the differential sensitivity of transiently transfected and integrated reporters to the co-expressed dsRNA originates simply from their expression levels, we examined dsRNA effects on different amounts of transfected *Renilla* luciferase reporters. We did not observe a significant difference in the sensitivity of different amounts of transiently transfected reporter to the co-expressed dsRNA (Fig. S3A).

### dsRNA-mediated suppression occurs at the level of translation

**Figure 5:**
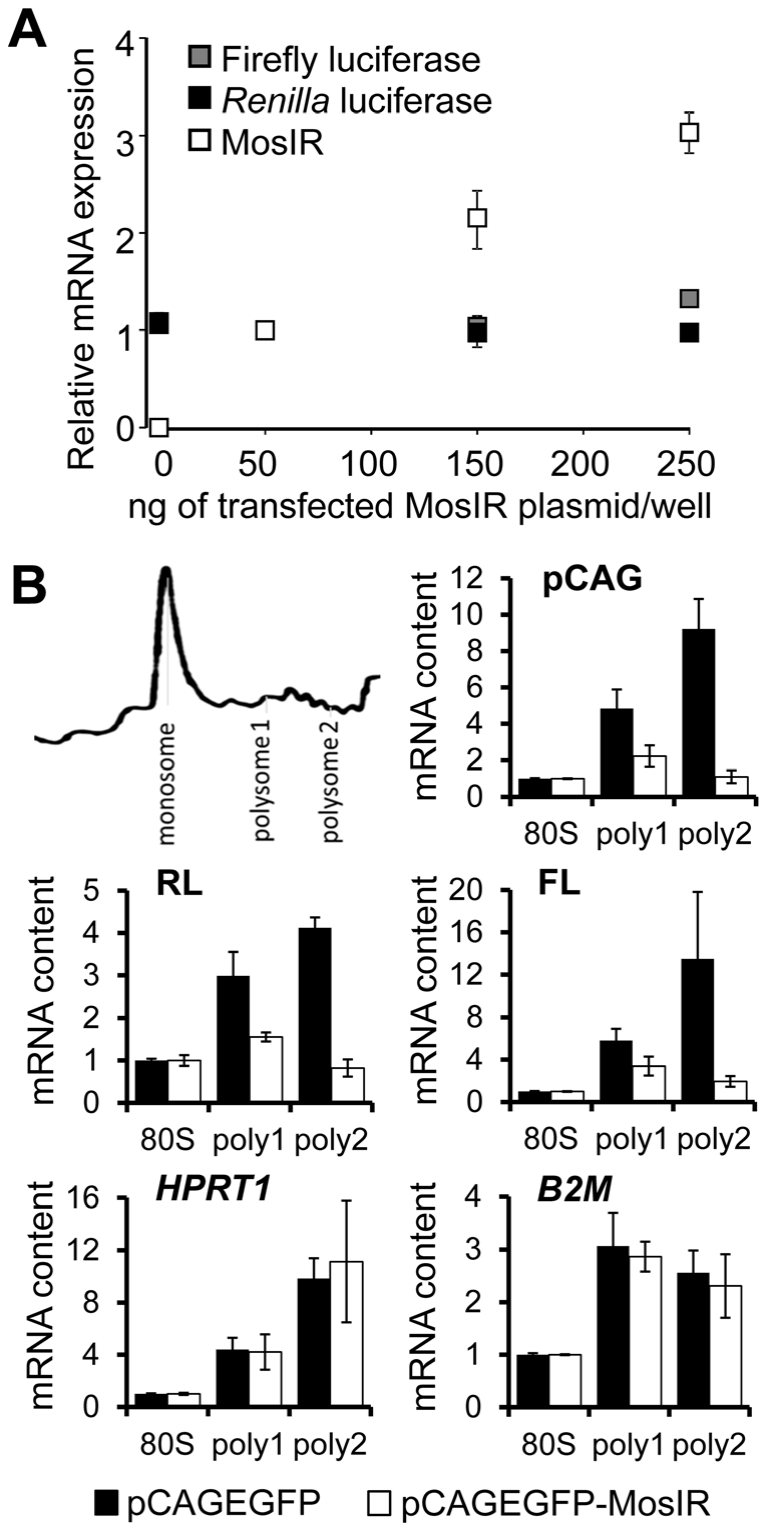
dsRNA inhibits translation of transcripts from transiently-transfected plasmids. (A) Transiently transfected RL and FL reporters are not inhibited at transcript levels. HEK-293 cells were transiently transfected with FL and RL reporters (100 ng/well) and increasing doses of pCAGEGFP-MosIR (0–250 ng/well). Amount of mRNA was analyzed by real-time PCR. Expression was normalized to *HPRT1* housekeeping gene and expression levels in cells transfected with 50 ng of MosIR plasmid were set to 1. Error bars  =  SEM. (B) dsRNA-dependent inhibition of translation affects more transiently transfected plasmids than endogenous genes. HEK-293 cells were transfected with RL, FL, and either pCAGEGFP or pCAGEGFP-MosIR. Distribution of mRNA in fractions collected during polysome profiling was analyzed by real-time PCR. For each sample, a fraction representing monosomes (80S) and early (poly1) and late (poly2) polysomes (depicted on the scheme) was included in the quantification. Expression levels in polysome fractions of pCAGEGFP- (black bars) and pCAGEGFP-MosIR- (white bars) transfected cells are normalized to 80S fraction. Panels show expression profiles for endogenous genes (HPRT1 and B2M), plasmid-expressed transcripts (FL, RL) and either pCAGEGFP or pCAGEGFP-MosIR transcript (pCAG). B2M, β_2_ microglobulin; HPRT1, hypoxantine phosphoryltransferase; FL, firefly luciferase; RL, *Renilla* luciferase.

To get a mechanistic insight into the silencing phenomenon, we investigated what stage of reporter expression was affected. The suppression could take place at the level of plasmid DNA (entry into/exclusion from the nucleus, plasmid stability), transcription, or it could occur post-transcriptionally. We did not observe any effect at the level of co-transfected plasmid DNA (data not shown). Next, we examined steady-state levels of transcripts originating from co-transfected plasmids using real-time PCR. These results showed that steady state levels of transcripts from constant amounts of co-transfected luciferase reporters remained constant while the level of transcripts from pCAGEGFP-MosIR was rising proportionally to the amount of co-transfected pCAGEGFP-MosIR (Fig. 5A). These data show that the mechanism suppressing co-transfected reporters is post-transcriptional.

To analyze translational rates of luciferase reporter transcripts and endogenous mRNAs, we performed polysome profiling of HEK-293 cells co-transfected with luciferase reporters and either pCAGEGFP-MosIR or pCAGEGFP plasmids. We isolated RNA from fractions corresponding to monosomes (80S) and polysomes and analyzed the abundance of mRNA in these fractions using real-time PCR. As expected, pCAGEGFP-transfected cells showed enrichment of various mRNAs in polysomal fractions compared to the monosomal fraction (Fig. 5B). In contrast, reduced abundance of luciferase mRNAs in polysomal fractions was observed in pCAGEGFP-MosIR-transfected cells (Fig. 5B). Remarkably, while there was the enrichment of pCAGEGFP mRNA in polysomal fractions, the amount of pCAGEGFP-MosIR mRNA on polysomes was also relatively low, suggesting that pCAGEGFP-MosIR inhibits the translation of its own transcript. Interestingly, endogenous *HPRT1* and β-2 microglobulin (*B2M*) mRNAs remained abundant in polysome fractions regardless of co-transfected plasmid. These data support the notion that suppression selectively inhibits all co-transfected reporters, while endogenous mRNAs (represented by *HPRT1* and *B2M*) remain efficiently translated.

### dsRNA-mediated suppression is PKR-dependent

**Figure 6:**
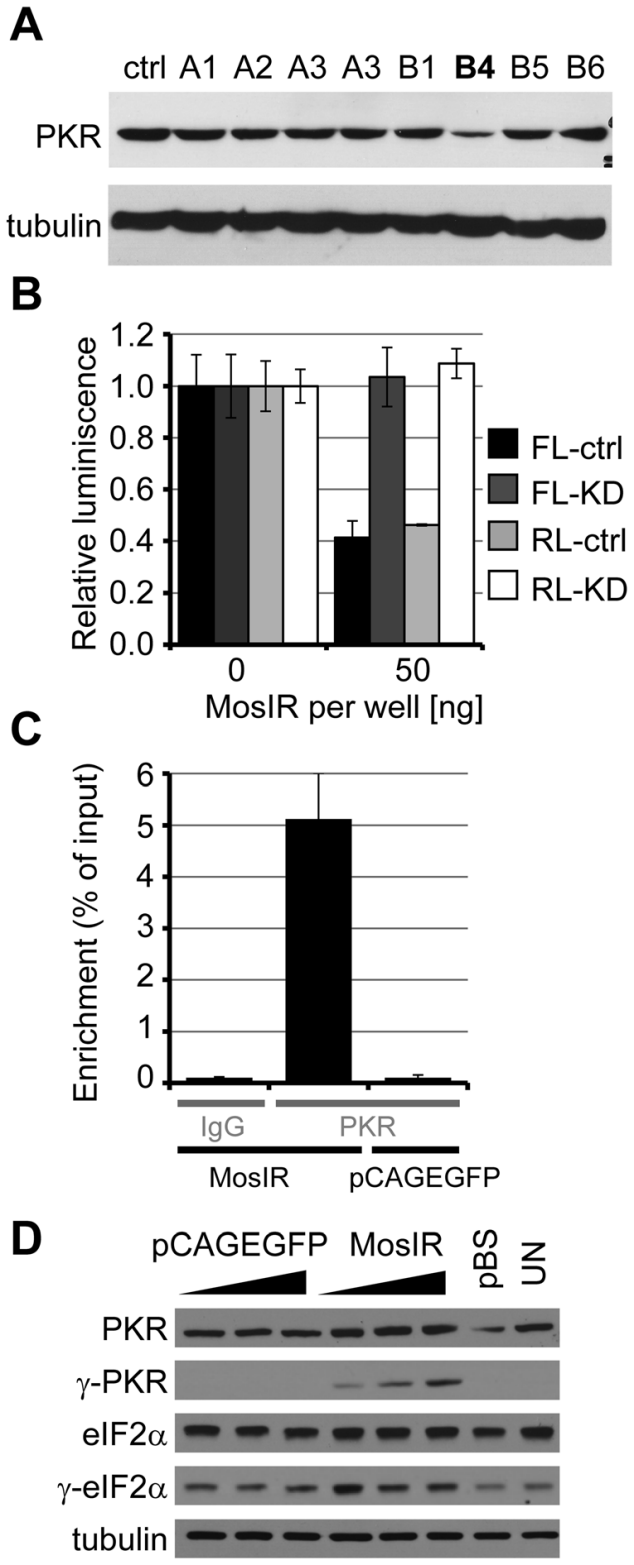
The inhibition of luciferase reporter activity is partly dependent on PKR protein level. (A) Stable cell line with shRNA-mediated PKR knock-down. Western blot analysis of HeLa stable cell lines carrying shRNA vector targeting PKR. Ctrl, parental HeLa cells; A1-B6, stably-transfected independent clones carrying antibiotic resistance. Clone B4 showed the highest PKR knock-down and was used for subsequent experiments. (B) Parental HeLa cells (ctrl) or HeLa cells with stably down-regulated PKR expression (KD) were co-transfected with 100 ng/well of each RL (light colors) and FL (dark colors) reporter plasmids and 0 or 50 ng/well of pCAGEGFP-MosIR. Parental plasmid pCAGEGFP was used to maintain a constant amount of transfected DNA. Data are shown as an average of two independent experiments performed in quadruplicates; Error bars  =  SEM. (C) pCAGEGFP-MosIR transcript associates with PKR. HEK-293 cells were transfected with pCAGEGFP or pCAGEGFP-MosIR. Cell lysates (24 hours post-transfection) were used for immunoprecipitation with anti-PKR antibody or control IgG antibody. Immunoprecipitated RNA was reverse-transcribed and used for real-time PCR analysis. Data are displayed as a percentage of input. Shown is the average of two experiments. Error bars  =  range of values. (D) pCAGEGFP-MosIR expression activates PKR. Western blotting analysis of HEK-293 cells transfected with increasing amount of pCAGEGFP or pCAGEGFP-MosIR (50–150–250 ng per well). pBluescript was added to maintain the amount of transfected DNA constant. pBS, pBluescript only. UN, untransfected cells; MosIR, pCAGEGFP-MosIR.

In the search for the mechanism inhibiting co-transfected luciferase reporters, we evaluated the possible role of PKR. We generated a stable PKR knockdown in HeLa cells by expressing shRNA targeting *Pkr* mRNA and selected the clone with the highest PKR downregulation (Fig. 6A) for further analysis. Transient transfection of the PKR knockdown cell line with pCAGEGFP-MosIR plasmid revealed relief of repression of luciferase reporters (Fig. 6B) indicating PKR dependence. A similar PKR dependence was also observed in HEK293 cells and for previously described pEGFP-C1-dependent translational inhibition (Fig. S4); in the latter case dsRNA originated from convergent transcription occurring within the plasmid backbone [9].

As PKR activation involves direct dsRNA binding to dsRNA-binding domain of PKR, we tested whether PKR directly binds dsRNA expressed from pCAGEGFP-MosIR by RNA immunoprecipitation followed by real-time PCR analysis. The plasmid-derived RNA was significantly enriched in pCAGEGFP-MosIR-transfected sample but not in the control (pCAGEGFP-transfected) sample (Fig. 6C). This suggests that the MosIR hairpin is directly bound by PKR. Accordingly, we detected increased PKR phosphorylation exclusively in pCAGEGFP-MosIR-transfected cells, in a concentration-dependent manner (Fig. 6D). The phosphorylation of eIF2α, a main PKR substrate, was marginally increased in pCAGEGFP-MosIR-transfected cells, consistent with limited effects on translation. As expected, phosphorylated PKR was not detected in pCAGEGFP-treated cells and control cells (Fig. 6D).

**Figure 7:**
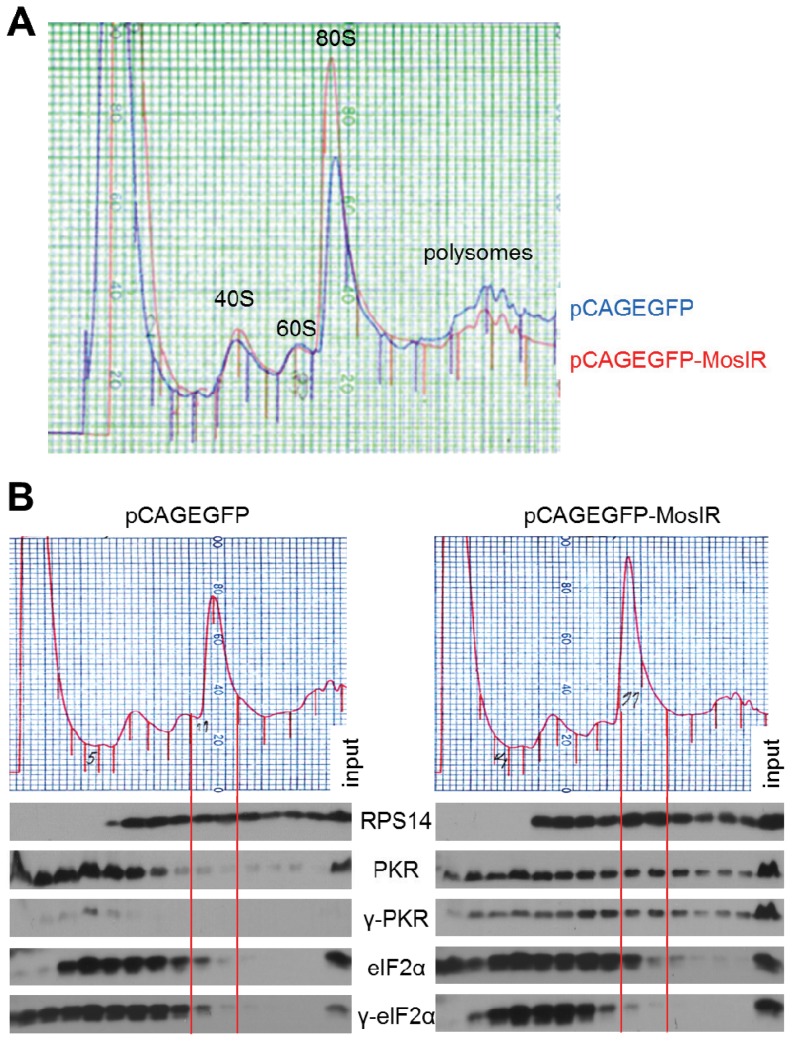
pCAGEGFP-MosIR expression affects the distribution of PKR and its phosphorylated form in polyribosome analysis. (A) Overlay of polysome profiles from HEK-293 cells transfected with either pCAGEGFP or pCAGEGFP-MosIR. (B) Polysome profile (upper part) and western blotting analysis (lower part) of respective fractions. The alignment of fractions on polysome profile and lanes on western blotting is highlighted by solid red lines flanking fractions 11 and 12. HEK-293 cells transfected with either pCAGEGFP or pCAGEGFP-MosIR were subjected to polysome profiling. Proteins were isolated from each fraction by ethanol precipitation and the same volume aliquots from each fraction were analyzed by polyacrylamide gel electrophoresis and western blotting. The last lane in each sample represents 1% of input. γ, phosphorylated form of protein; RPS14, ribosomal protein S14. The experiment was repeated three times, a representative result is shown.

Finally, we examined PKR distribution along polysome profiles of HEK-293 cells. Expression of long dsRNA resulted in a reproducibly increased amount of monosomes/free ribosomes and a reduced amount of polysomes (Fig. 7A), indicating a modest inhibition of translation initiation, which in principle could be attributed to partially increased levels of eIF2α phosphorylation. Distribution of PKR and its phosphorylated form along the polysome profile differed between cells transfected with pCAGEGFP-MosIR or pCAGEGFP (Fig. 7B). This is apparent when compared with the distribution of the ribosomal protein S14 (RPS14), which should reflect the amount of ribosomes in individual fractions. Consistent with the higher monosome peak in polysome profiles of cells transfected with pCAGEGFP-MosIR, we observed apparently higher abundance of RPS14 in monosomal fractions (framed by solid red lines in Fig. 7B) suggesting that dsRNA expression leads to an accumulation of monosomes (Fig. 7B). Phosphorylated PKR was negligible in all pCAGEGFP fractions, consistent with its absence in the unfractionated lysate (Fig. 7B). In cells transfected with pCAGEGFP-MosIR, both PKR and its phosphorylated form were redistributed from soluble to monosomal and polysomal fractions. This could reflect PKR redistribution associated with translational inhibition or a direct binding of PKR to the translated MosIR transcript. Notably, the distribution of eIF2α and its naturally phosphorylated form was affected only marginally (Fig. 7B).

## Discussion

Our results provide a framework for several previously reported phenomena, namely 1) negative correlation between plasmid-derived translation and the amount of PKR [7], [20], [21], 2) enhanced plasmid expression after treatment with PKR inhibitors [20], [22], [23], 3) restricted PKR effects and selective inhibition of specific mRNAs [6], [7], and 4) dsRNA-induced sequence-independent suppression of transiently-transfected plasmid reporters [24], [25]. We show that expressed dsRNA induces unique PKR-dependent translational repression that is strongly biased towards transcripts from transiently transfected plasmids. The molecular mechanism of this specific repression does not seem to involve the typical IFN response; a possible contribution of eIF2α-phosphorylation remains to be elucidated. The fact that presumably the same effect was observed on numerous occasions also without intentional expression of dsRNA (i.e. when using plasmids not previously known to produce dsRNAs) can be explained by the complex transcription of transfected plasmids, which employs multiple cryptic promoters and thus has potential to generate dsRNA. We previously reported an example where a common plasmid indeed produces dsRNA because of convergent transcription in a neomycin/kanamycin cassette and causes the suppression of co-transfected reporters [9], which is also PKR-dependent (Fig. S4). Sequence-independent repression of transiently transfected reporters was also shown for different types of RNA hairpins [24], [25] although a formal proof that the same mechanisms was responsible for the effect as the one described here was not provided. In any case, available data indicate that sequence-independent silencing can be induced by different types of expressed dsRNA, i.e. it is not specific for the dsRNA-bearing translatable mRNA produced by pCAGEGFP-MosIR and that it occurs in different cell types. So far, we documented the PKR-dependent silencing phenomenon for HEK-293 and HeLa cells. We observed sequence-independent reporter inhibition induced by dsRNA expression also in 3T3 cells, P19 and HepG2 cells (unpublished observations). It is possible that this silencing phenomenon is common to mammalian somatic cell types expressing PKR and that many of the sequence-independent effects observed upon dsRNA expression in mammalian cells were its manifestations. However, it is important to point out that dsRNA could induce silencing of co-transfected reporters through different mechanisms in *cis* or *trans* in mammalian cells, thus one should be cautious when interpreting dsRNA effects in transiently transfected cells. In addition to the phenomenon described here, dsRNA can affect a reporter in *trans* in a sequence-dependent manner through RNAi or in a sequence-independent manner through the interferon response. *Cis* mechanisms might involve cleavage by Dicer, Staufen-mediated mRNA decay [26], various *cis* effects of adenosine deamination [27], or translational repression [28]. While inhibition of luciferase reporters is most likely occurring in *trans* (via dsRNA-activated PKR), pCAGEGFP-MosIR could be affected by the same *trans* effect as well as *cis* effect similar to those induced by inverted Alu dsRNA reported by Capshew et al. [28].

The most remarkable feature of the silencing of co-transfected reporters is that cells somehow distinguish between transcripts originating from a transiently-transfected plasmid and a plasmid stably integrated in the genome (Fig. 4D). Plasmid expression could be more sensitive to a dsRNA-responding pathway than expression originating from the genome, as has been reported by Gommans and Maas, who showed that ADAR1 can enhance plasmid-based expression at the transcriptional level [29]. However, there is most likely no mechanistical connection with the post-transcriptional phenomenon reported here. In any case, our observations are consistent with Terenzi *et al*., who described that expression from non-viral and viral vectors is suppressed in mammalian cells in a PKR-dependent manner while translation of endogenous proteins is not strongly affected [20]. One possibility is that plasmid-borne transcripts are somehow differentiated either by RNA properties (such as poly(A) length or covalent modifications) or by different protein factors bound to these transcripts. Another possibility is that the sensitivity is consequential; dsRNA appearance in a co-transfection experiment could make newly synthesized mRNAs more prone to translational repression. Indeed, PKR inhibition was demonstrated to stimulate translation of newly synthesized mRNA [22]. Our preliminary experiments would favor the latter option since plasmid-borne transcripts appear not to be dsRNA sensitive when reporter plasmids are transfected 24 hours before the dsRNA-expressing plasmid (Fig. S3B). However, this hypothesis needs further testing using different types of dsRNA and inducible expression of dsRNA allowing for precise timing of dsRNA appearance during expression of luciferase reporters.

As aforementioned, the exact role of PKR in the selective repression of co-transfected reporters remains to be defined. While PKR binds the expressed dsRNA and becomes phosphorylated, it does not activate the typical response marked by global robust repression of translation and activation of interferon-stimulated genes in HEK-293 and HeLa cells transfected with pCAGEGFP-MosIR [8]. It is possible that the transformed cell lines, which have been in cultured for decades, may represent an atypical case of partial PKR activation disconnected from the interferon response. However, normal development and appearance of transgenic mice carrying an active pCAGEGFP-MosIR transgene rather suggests, that nuclear dsRNA expression is not a strong inducer of the interferon response. At the same time, the expression of long dsRNA in HEK-293 and HeLa cells partially inhibited translation initiation as it increased amounts of monosomes/free ribosomes and reduced amounts of polysomes (Fig. 7A). It is possible that highly abundant transcripts from co-transfected plasmids [9] occupy a considerable portion of translation machinery; thus, reducing the pool of free factors for cellular mRNA translation that is otherwise not specifically inhibited. Alternatively, cellular translation is partially affected but only to the extent that does not grossly affect cell viability and has a minimal impact on translation of cellular mRNAs (Fig. 5B) and on the transcriptome [8]. A potential valuable lead for future experiments is our novel observation that phosphorylated PKR associates with high polysomes upon transfection of pCAGEGFP-MosIR (Fig. 7B). Whether this reflects a general aspect of PKR role in translational inhibition or the PKR binding to the presumably abundant translated MosIR transcript is currently under investigation.

In our view, supported by previous studies of processing of MosIR transcripts [8], [30], dsRNA appearing during transient transfection represents a unique case of nuclear dsRNA expression, where dsRNA can enter multiple pathways at the same time, but the dominating effect is PKR-dependent repression of co-transfected reporters occurring in the absence of the interferon response. It has been demonstrated that dsRNA expressed from a plasmid can induce the interferon response [31]. We explain different reported effects of expressed dsRNA on the activation of the interferon response by the existence of a threshold, which depends on a cell type and the particular dsRNA molecule and which was not reached in our experiments where dsRNA was either a part of a translatable spliced mRNA ([8] and data reported here) or originated from a spurious transcription from the plasmid backbone [9]. The existence of the threshold is conceivable considering survival of mammalian cells whose genomes have a high potential to express dsRNA [32], [33].

Taken together, our results show that expressed dsRNA can lead to PKR activation resulting in a limited repression of cellular translation. At the same time, the translation of transiently-transfected reporters is exceptionally sensitive to this PKR-dependent selective translation repression. While the physiological role of this phenomenon is unclear, it has apparent features of a defense response against expression of foreign DNA. In any case, our findings are mainly important from the methodological perspective, since dsRNA expression can be a potent source of artifacts in transient transfection experiments, which are frequently used for studying gene function in cultured cells. Transcription of transfected plasmids is very complex and it is difficult to predict whether or not a plasmid will produce dsRNA affecting co-transfected reporters.

A transient transfection experiment frequently involves three components: inducer, target, and control. A dual luciferase assay provides a convenient pair of target and control reporters, where one luciferase (target) is directly affected by the inducer, and the non-targeted luciferase (control) is used for normalizing values of the target. Therefore, to minimize the risk of artifacts in co-transfection experiments, we recommend to carefully investigate each case where the control reporter consistently exhibits reduced expression when co-transfected with a particular inducer plasmid. dsRNA-mediated suppression of the reporter would manifest as an inducer of dosage-dependent difference between control reporter transcript and protein levels. In addition, one should design highly similar inducer and control plasmids– e.g. it is better to compare a particular protein expression vector with a vector expressing a catalytically dead or deletion mutant rather than using just an empty vector. The rationale is that each sequence is a potential source of cryptic promoters, which may produce convergent transcription, so the more similar the plasmids co-transfected with reporters are, the less likely one of them would produce a significantly higher amount of dsRNA, which would bias the sample transfected with that plasmid. Importantly, different cell types do not exhibit the same sensitivity to dsRNA-mediated repression of co-transfected reporters. For example, dsRNA-induced repression of reporters in mouse 3T3 cells did not exceed 50% (Fig. 2B). Therefore, mouse 3T3 cells might be a better choice for transient transfection experiments than commonly used HEK-293 and HeLa cells, in which transiently transfected reporters are highly sensitive to dsRNA. Finally, it is advisable to validate results from transient co-transfections using a model system where gene expression originates from the genomic context or using a serial transfection where the inducer and reporters are transfected separately. However, suitable use of serial transfection would be limited to situations where one can either achieve high transfection efficiency or can separate doubly-transfected cells for analysis.

## Supporting Information

Figure S1 **pCAGEGFP-Lin28IR and pCAGEGFP-Elavl2IR vectors produce dsRNA.** (A, B) dsRNA was detected as described previously [8] by amplifying RNase-T1 resistant RNA from lysates of cells transfected with pCAGEGFP-Lin28IR or pCAGEGFP-Elavl2IR. Briefly, HEK293 cells were transfected with either pCAGEGFP-Lin28IR (A) or pCAGEGFP-Elavl2IR (B) plasmid. Cells were harvested 24 hours post-transfection and divided into three parts: 1) untreated (input), 2) treated with RNase T1 (RNase T1), and 3) denatured before RNase T1 treatment (heat+RNase T1). The presence of dsRNA was analyzed by reverse transcription and real-time PCR performed in a triplicate. Data are shown as relative average amplification of dsRNA region compared to 10% input, which was set to 1. (C) dsRNA immunoprecipitation. HEK293 cells were transfected with pCAGEGFP-Elavl2IR, pCAGEGFP-Lin28IR, pCAGEGFP-MosIR or parental pCAGEGFP plasmid. After 24 hours, cells were lysed and dsRNA was immunoprecipitated using J2 antibody [34] using the same protocol as for PKR immunoprecipitation shown in Fig. 6C. Immunoprecipitated RNA was isolated, reverse-transcribed, and analyzed by real-time PCR in a triplicate using primers common for all plasmids. Data are shown as an average enrichment in samples transfected with hairpin-expressing plasmids relative to pCAGEGFP plasmid.
(TIFF)

Figure S2 **Analysis of IL-8 expression upon pCAGEGFP-MosIR transfection.** HEK293 cells were transfected in triplicates with 0–250 ng of pCAGEGFP-MosIR or pCAGEGFP plasmid per well in 24-well plate; the total amount of transfected DNA was kept constant by adding pBluescript plasmid. After 48 hours, RNA was isolated, reverse-transcribed, and analyzed by real-time PCR. *IL-8* repression was normalized to *HPRT1* housekeeping gene and expression level in cells transfected with pBluescript (BS) plasmid only was set to 1. Error bars  =  SEM.
(TIFF)

Figure S3 **Reporter dilution and serial transfection.** (A) Reporter dilution. HEK293 cells were transiently transfected with various concentrations of *Renilla* reporter (2.5 – 125 ng/well in 24-well plate) and indicated amounts of either pCAGEGFP-MosIR plasmid (triangles) or control pCAGEGFP-MosMos plasmid (squares). The total amount of DNA was kept constant by adding pBluescript. Luciferase activity was analyzed 48 hours after transfection. Relative luciferase activity is shown; activities in samples transfected with 0 ng of pCagEGFP-MosIR/pCagEGFP-MosMos were set to 1. Data represent one experiment performed in a triplicate transfection. Error bars  =  SEM. (B) Serial transfection. HEK-293 cells were transiently transfected in triplicates with *Renilla* and firefly luciferase reporters. After 24 hours, cells were transfected with indicated amounts of pCAGEGFP-MosIR or pCAGEGFP plasmids. The total amount of DNA was kept constant by adding pBluescript. Luciferase activity was analyzed 48 hours after the second transfection. Activities in samples transfected with 0 ng of pCAGEGFP-MosIR/pCAGEGFP were set to 1. Error bars  =  SEM.
(TIFF)

Figure S4 **PKR-dependent reporter downregulation.** (A) PKR-dependent reporter downregulation in HEK-293 cells. Parental HEK-293 cells (ctrl) or HEK-293 cells with stably downregulated PKR expression (KD) were co-transfected in triplicates with firefly (FL) or Renilla (RL) luciferase reporters (100 ng/well in 24-well plate each) together with 0 or 50 ng of pCAGEGFP-MosIR plasmid; pCAGEGFP was used to maintain constant amount of DNA. Luciferase activity in cells transfected with pCAGEGFP only was set to 1. Error bars  =  SEM. (B) Parental HeLa cells (ctrl) or HeLa cells with stably downregulated PKR expression (KD) were co-transfected in triplicates with firefly (FL) or *Renilla* (RL) luciferase reporters (100 ng/well in 24-well plate each) together with 0 or 50 ng of pEGFP-C1 plasmid; pBluescript was used to maintain constant amount of DNA. Luciferase activity in cells transfected with pBluescript only was set to 1. Error bars  =  SD.
(TIFF)

Table S1 **Primer sequences.**
(DOCX)

## References

[1] Gantier MP, Williams BR (2007) The response of mammalian cells to double-stranded RNA. Cytokine Growth Factor Rev 18: 363–371. 17698400 10.1016/j.cytogfr.2007.06.016 PMC2084215

[2] Carthew RW, Sontheimer EJ (2009) Origins and Mechanisms of miRNAs and siRNAs. Cell 136: 642–655. 19239886 10.1016/j.cell.2009.01.035 PMC2675692

[3] Nishikura K (2010) Functions and regulation of RNA editing by ADAR deaminases. Annu Rev Biochem 79: 321–349. 20192758 10.1146/annurev-biochem-060208-105251 PMC2953425

[4] Sadler AJ, Williams BR (2008) Interferon-inducible antiviral effectors. Nat Rev Immunol 8: 559–568. 18575461 10.1038/nri2314 PMC2522268

[5] Sadler AJ, Williams BR (2007) Structure and function of the protein kinase R. Curr Top Microbiol Immunol 316: 253–292. 17969452 10.1007/978-3-540-71329-6_13

[6] Ben-Asouli Y, Banai Y, Pel-Or Y, Shir A, Kaempfer R (2002) Human interferon-gamma mRNA autoregulates its translation through a pseudoknot that activates the interferon-inducible protein kinase PKR. Cell 108: 221–232. 11832212 10.1016/s0092-8674(02)00616-5

[7] Kaufman RJ, Davies MV, Pathak VK, Hershey JW (1989) The phosphorylation state of eucaryotic initiation factor 2 alters translational efficiency of specific mRNAs. Mol Cell Biol 9: 946–958. 2657393 10.1128/mcb.9.3.946-958.1989 PMC362683

[8] Nejepinska J, Malik R, Filkowski J, Flemr M, Filipowicz W, et al (2012) dsRNA expression in the mouse elicits RNAi in oocytes and low adenosine deamination in somatic cells. Nucleic Acids Res 40: 399–413. 21908396 10.1093/nar/gkr702 PMC3245926

[9] Nejepinska J, Malik R, Moravec M, Svoboda P (2012) Deep sequencing reveals complex spurious transcription from transiently transfected plasmids. PLoS One 7: e43283. 22916237 10.1371/journal.pone.0043283 PMC3420890

[10] Kaname T, Huxley C (2001) Simple and efficient vectors for retrofitting BACs and PACs with mammalian neoR and EGFP marker genes. Gene 266: 147–153. 11290429 10.1016/s0378-1119(01)00375-4

[11] Sambrook J, Russell DW (2006) Calcium-phosphate-mediated Transfection of Eukaryotic Cells with Plasmid DNAs. CSH Protoc 2006. 10.1101/pdb.prot3871 22485343

[12] van de Wetering M, Oving I, Muncan V, Pon Fong MT, Brantjes H, et al (2003) Specific inhibition of gene expression using a stably integrated, inducible small-interfering-RNA vector. EMBO Rep 4: 609–615. 12776180 10.1038/sj.embor.embor865 PMC1319205

[13] Valasek L, Szamecz B, Hinnebusch AG, Nielsen KH (2007) In vivo stabilization of preinitiation complexes by formaldehyde cross-linking. Methods Enzymol 429: 163–183. 17913623 10.1016/S0076-6879(07)29008-1

[14] Stein P, Svoboda P, Schultz RM (2003) Transgenic RNAi in mouse oocytes: a simple and fast approach to study gene function. Dev Biol 256: 187–193. 12654301 10.1016/s0012-1606(02)00122-7

[15] Stein P, Zeng F, Pan H, Schultz RM (2005) Absence of non-specific effects of RNA interference triggered by long double-stranded RNA in mouse oocytes. Dev Biol 286: 464–471. 16154556 10.1016/j.ydbio.2005.08.015

[16] Svoboda P, Stein P, Filipowicz W, Schultz RM (2004) Lack of homologous sequence-specific DNA methylation in response to stable dsRNA expression in mouse oocytes. Nucleic Acids Res 32: 3601–3606. 15247344 10.1093/nar/gkh697 PMC484184

[17] Svoboda P, Stein P, Schultz RM (2001) RNAi in mouse oocytes and preimplantation embryos: effectiveness of hairpin dsRNA. Biochem Biophys Res Commun 287: 1099–1104. 11587535 10.1006/bbrc.2001.5707

[18] Colledge WH, Carlton MB, Udy GB, Evans MJ (1994) Disruption of c-mos causes parthenogenetic development of unfertilized mouse eggs. Nature 370: 65–68. 8015609 10.1038/370065a0

[19] Hashimoto N, Watanabe N, Furuta Y, Tamemoto H, Sagata N, et al (1994) Parthenogenetic activation of oocytes in c-mos-deficient mice. Nature 370: 68–71. 8015610 10.1038/370068a0

[20] Terenzi F, deVeer MJ, Ying H, Restifo NP, Williams BR, et al (1999) The antiviral enzymes PKR and RNase L suppress gene expression from viral and non-viral based vectors. Nucleic Acids Res 27: 4369–4375. 10536144 10.1093/nar/27.22.4369 PMC148718

[21] Wang Y, Samuel CE (2009) Adenosine deaminase ADAR1 increases gene expression at the translational level by decreasing protein kinase PKR-dependent eIF-2alpha phosphorylation. J Mol Biol 393: 777–787. 19733181 10.1016/j.jmb.2009.08.070 PMC2763985

[22] Rubin CM, Kimura RH, Schmid CW (2002) Selective stimulation of translational expression by Alu RNA. Nucleic Acids Res 30: 3253–3261. 12136107 10.1093/nar/gkf419 PMC135740

[23] Kaufman RJ, Murtha P (1987) Translational control mediated by eucaryotic initiation factor-2 is restricted to specific mRNAs in transfected cells. Mol Cell Biol 7: 1568–1571. 3600637 10.1128/mcb.7.4.1568-1571.1987 PMC365249

[24] Yang S, Tutton S, Pierce E, Yoon K (2001) Specific double-stranded RNA interference in undifferentiated mouse embryonic stem cells. Mol Cell Biol 21: 7807–7816. 11604515 10.1128/MCB.21.22.7807-7816.2001 PMC99950

[25] Paddison PJ, Caudy AA, Hannon GJ (2002) Stable suppression of gene expression by RNAi in mammalian cells. Proc Natl Acad Sci U S A 99: 1443–1448. 11818553 10.1073/pnas.032652399 PMC122210

[26] Gong C, Tang Y, Maquat LE (2013) mRNA-mRNA duplexes that autoelicit Staufen1-mediated mRNA decay. Nat Struct Mol Biol 20: 1214–1220. 24056942 10.1038/nsmb.2664 PMC3947523

[27] DeCerbo J, Carmichael GG (2005) Retention and repression: fates of hyperedited RNAs in the nucleus. Curr Opin Cell Biol 17: 302–308. 15901501 10.1016/j.ceb.2005.04.008

[28] Capshew CR, Dusenbury KL, Hundley HA (2012) Inverted Alu dsRNA structures do not affect localization but can alter translation efficiency of human mRNAs independent of RNA editing. Nucleic Acids Res 40: 8637–8645. 22735697 10.1093/nar/gks590 PMC3458544

[29] Gommans WM, Maas S (2008) Characterization of ADAR1-mediated modulation of gene expression. Biochem Biophys Res Commun 377: 170–175. 18835380 10.1016/j.bbrc.2008.09.109

[30] Flemr M, Malik R, Franke V, Nejepinska J, Sedlacek R, et al (2013) A retrotransposon-driven dicer isoform directs endogenous small interfering RNA production in mouse oocytes. Cell 155: 807–816. 24209619 10.1016/j.cell.2013.10.001

[31] Gantier MP, Baugh JA, Donnelly SC (2007) Nuclear transcription of long hairpin RNA triggers innate immune responses. J Interferon Cytokine Res 27: 789–797. 17892400 10.1089/jir.2006.0152

[32] Katayama S, Tomaru Y, Kasukawa T, Waki K, Nakanishi M, et al (2005) Antisense transcription in the mammalian transcriptome. Science 309: 1564–1566. 16141073 10.1126/science.1112009

[33] Svoboda P, Di Cara A (2006) Hairpin RNA: a secondary structure of primary importance. Cell Mol Life Sci 63: 901–908. 16568238 10.1007/s00018-005-5558-5 PMC11136179

[34] Schonborn J, Oberstrass J, Breyel E, Tittgen J, Schumacher J, et al (1991) Monoclonal antibodies to double-stranded RNA as probes of RNA structure in crude nucleic acid extracts. Nucleic Acids Res 19: 2993–3000. 2057357 10.1093/nar/19.11.2993 PMC328262

