# Supplementary material for: Reporters Transiently Transfected into Mammalian Cells Are Highly Sensitive to Translational Repression Induced by dsRNA Expression
Source: PLoS One. 2014 Jan 27;9(1):e87517. doi: 10.1371/journal.pone.0087517 (PMC3903663; doi:10.1371/journal.pone.0087517)
Supplement: Table S1 — Primer sequences. (DOCX) [file pone.0087517.s005.docx]

**Table S1 Primer sequences**

| **Primer name** | **sequence** | **used for** |
| --- | --- | --- |
| Mos3+IR RT.rev | GCCAGAAGTCAGATGCTCAAGG | reverse transcription (Fig S1) |
| FL_Fwd | GCTACAAACGCTCTCATCGACAAG | qPCR-FL (Fig. 5) |
| FL_Rev | GTATTTGATCAGGCTCTTCAGCCG | qPCR-FL (Fig. 5) |
| RL_Fwd | CAGATTGTCCGCAACTACAACGCC | qPCR-FL (Fig. 5) |
| RL_Rev | CTTACCCATTTCATCTGGAGCGTC | qPCR-FL (Fig. 5) |
| huB2MG_Fwd | GTATGCCTGCCGTGTGAACCATC | qPCR-FL (Fig. 5) |
| huB2MG_Rev | CAAATGCGGGCATCTTCAAACCTCC | qPCR-FL (Fig. 5) |
| pCag.fwd3a | GGCTCTGACTGACCGCGTTAC | qPCR-CAG (Fig. 5, 6C, S1C) |
| EGFP.rev.seq | GCTGAACTTGTGGCCGTTTACG | qPCR-CAG (Fig. 5, 6C, S1C) |
| huIL-8_Fwd | CRGCAGCTCTGTGTGAAGGTGC | qPCR-IL-8 (Fig. S2) |
| huIL-8_Rev | CAGTTTTCCTTGGGGTCCAGACAG | qPCR-IL-8 (Fig. S2) |
| Lin28.3523.fwd | GAAGGCTGTGTGATATTTCCCTTG | RNase T1 (Fig. S1) |
| Lin28.3692.rev | GCTGCTCTCTGTGGACCACTATTC | RNase T1 (Fig. S1) |
| Elavl2.3112.fwd | GAACACAGAGGACAGCAAGACCAA | RNase T1 (Fig. S1) |
| Elavl2.3273.rev | TTCACAAATCCGTAACCCAAGCTC | RNase T1 (Fig. S1) |
| huHPRT_Fwd | TGACCTTGATTTATTTTGCATACC | housekeeping gene (Fig. 5A,S2) |
| huHPRT_Rev | CGAGCAAGACGTTCAGTCCT | housekeeping gene (Fig. 5A,S2) |
